# Event-related potentials in a human serial conditioning paradigm

**DOI:** 10.1192/j.eurpsy.2021.504

**Published:** 2021-08-13

**Authors:** Y. Pavlov, B. Kotchoubey

**Affiliations:** 1 Department Of Psychology, Ural Federal University, Ekaterinburg, Russian Federation; 2 Institute Of Medical Psychology, University of Tuebingen, Tuebingen, Germany

**Keywords:** ERP, Fear conditioning, EEG

## Abstract

**Introduction:**

In a serial compound conditioning paradigm, a sequence of several conditioned stimuli (CS) is predictive to an unconditioned stimulus (US) (e.g., CSA->CSB->US). Animal research showed that, when the US is aversive, CSA elicits the strongest conditioned response, while CSB appears redundant. These effects of primacy and proximity have never been investigated in humans.

**Objectives:**

To study the effects of temporal proximity of imminent threat and safety in serial compound conditioning.

**Methods:**

Twenty-two participants were presented with sequences [CSA->CSB->CSC->CSD]. In 55 trials all four CS were identical vowels (e.g, [oh]), and no US was presented. In the other 55 trials, the CSA was different (CSA+, e.g., [uh]), and the CSD was followed by an electrical shock (US) 2.5 times higher than the individual pain threshold.

**Results:**

No ERP component distinguished between CS- and CS+ for the first three stimuli in the sequence (i.e., CSA, CSB, CSC). The last CS (CSD) elicited a strong fronto-central CNV only when it was followed by US. Moreover, already after the CSA- (which signalized that no shock would be presented on that trial) the power of alpha oscillations over the somatosensory cortex significantly increased, particularly on the side contralateral to the hand that was electrically stimulated on US trials. The alpha increment lasted up to the onset of the US.

**Conclusions:**

The data indicate two possible mechanisms of adjustment to predictable threat, one of which relies on safety signals (manifested in alpha increment), and the other is related to flight response (manifested in the CNV immediately preceding the shock).

